# Content of Fatty Acid and Eicosanoids in Muscle and Intestinal Tissue of C57BL/6 Mice Subjected to Long-Term Caloric Restriction

**DOI:** 10.3390/nu18030518

**Published:** 2026-02-03

**Authors:** Joanna Palma, Karolina Skonieczna-Żydecka, Dominika Maciejewska-Markiewicz, Katarzyna Zgutka, Katarzyna Piotrowska, Ewa Stachowska

**Affiliations:** 1 Department of Biochemical Science, Pomeranian Medical University in Szczecin, Broniewskiego 24, 71-460 Szczecin, Poland; joanna.palma@pum.edu.pl (J.P.); karolina.skonieczna.zydecka@pum.edu.pl (K.S.-Ż.); 2 Department of Human Nutrition and Metabolomics, Pomeranian Medical University in Szczecin, Broniewskiego 24, 71-460 Szczecin, Poland; dominika.maciejewska@pum.edu.pl; 3 Department of Physiology in Health Sciences, Faculty of Health Sciences, Pomeranian Medical University, Żołnierska 48, 70-210 Szczecin, Poland; katarzyna.zgutka@pum.edu.pl; 4 Department of Physiology, Pomeranian Medical University, 70-111 Szczecin, Poland; katarzyna.piotrowska@pum.edu.pl

**Keywords:** caloric restriction (CR), fatty acids derivatives, intestines, muscle, C57BL/6 mouse

## Abstract

Background: Caloric restriction (CR) is a dietary intervention based on limiting calories relative to the basic energy needs of the organism, which changes the intensity of metabolism, causes changes in the functioning of the endocrine and sympathetic systems, and influences the expression of genes in muscle, heart, and brain cells. During the use of CR, there is a transition from carbohydrate supply to increased fat metabolism. Fatty acids are more or less susceptible to free radicals, depending on their molecular structure. Oxidation (peroxidation) contributes to the production of metabolites (including hydroxyeicosatetraenoic acid and hydroxyoctadecadienoic acid), some of which are involved in inflammation. Methods: The aim of this study was to evaluate the effects of long-term caloric restriction on the tissue levels of selected fatty acids and fatty acid-derived lipid mediators with pro-inflammatory or anti-inflammatory properties in skeletal muscle and intestinal tissues. The study was carried out on C57BL/6 mice. During the 8-month experiment, the mice in the study group were fed a 30% calorie restricted diet—according to the Every-Other-Day Diet concept. Analyses were performed on intestinal and muscle tissues collected from animals. Fatty acid derivatives were isolated using solid-phase extraction (C-18 columns) columns, and isolation of fatty acids was performed using a modified Folch method. The compounds were analyzed by liquid and gas chromatography. Results: CR induced detectable alterations in both fatty acid profiles and lipid mediator concentrations in a tissue-specific manner. However, most of these changes did not remain statistically significant after multiple testing correction. Conclusions: These findings suggest potential effects of long-term CR on lipid signaling pathways, although the current dataset lacks the statistical power required to draw definitive conclusions. This study highlights the need for further research using larger sample sizes and integrated multiomic approaches to elucidate the molecular mechanisms underlying lipidomic adaptations to prolonged caloric restriction.

## 1. Introduction

Obesity has emerged as a global health concern, primarily driven by improper dietary habits, a sedentary lifestyle, and insufficient physical activity [1,2]. Excessive body weight constitutes a major risk factor for the development of numerous metabolic disorders [3,4]. Despite significant advances in medical science and the emergence of novel therapeutic strategies aimed at weight reduction, obesity remains a persistent and growing public health issue [1,5]. For decades, extensive research has been conducted to identify an optimal and effective approach to counteract obesity and its associated complications. Importantly, advancing age is accompanied by substantial alterations in metabolic processes, including dysregulation of fatty acid and nucleic acid metabolism, as well as disruption of redox homeostasis manifested by an imbalance between pro-oxidant and anti-oxidant systems. These changes, when coupled with inadequate nutrition, further contribute to the progression of chronic diseases [3,6].

**Figure 1:**
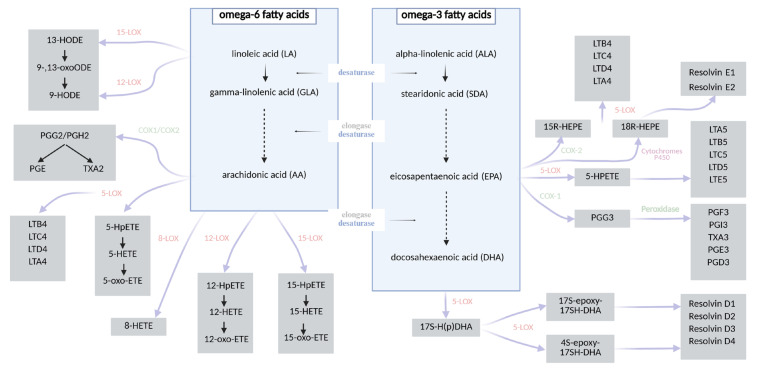
A chart showing the synthesis of fatty acid derivatives [27,28,29,30,39,40].

Caloric restriction (CR) constitutes one of the most extensively studied non-pharmacological strategies in the prevention and management of obesity and related metabolic disorders. CR typically involves a reduction in daily caloric intake by approximately 10–40% relative to the organism’s baseline energy requirements, without inducing malnutrition [7,8]. This intervention exerts systemic effects on metabolism, leading not only to significant reductions in body weight, but also to the deceleration of age-related pathologies and the activation of molecular pathways associated with longevity and cellular homeostasis [9,10,11,12,13,14,15,16]. Numerous studies have demonstrated that CR diminishes cellular proliferation, thereby attenuating the risk of tumorigenesis. Furthermore, it exerts beneficial effects on glucose homeostasis by enhancing insulin sensitivity in peripheral tissues, preventing insulin resistance and reducing the incidence of type 2 diabetes mellitus [9,11]. These effects are linked to a downregulation of hepatic gluconeogenesis, increased β-oxidation of fatty acids, and enhanced glycolytic activity. Metabolically, the organism adapts to energy limitation by mobilizing lipid reserves from white adipose tissue, thereby promoting metabolic flexibility [15,16,17,18,19,20]. Fatty acids, particularly polyunsaturated species, are essential modulators of cellular signaling and energy metabolism, yet, due to their structural susceptibility to oxidation, they are also primary targets of free radical-induced damage. Their peroxidation generates a range of bioactive lipid mediators, including isoprostanes and aldehydes, some of which play a key role in propagating inflammatory responses [11,16,21,22,23]. Oxidative stress, defined as an imbalance between the generation of reactive oxygen species (ROS) and the efficiency of anti-oxidant defense mechanisms, is a critical contributor to the pathogenesis of metabolic, cardiovascular, and neurodegenerative diseases. Elevated oxidative stress promotes mitochondrial dysfunction, endothelial damage, and lipid peroxidation and accelerates cellular senescence. Notably, CR has been shown to attenuate oxidative stress by reducing ROS production, enhancing anti-oxidant enzyme activity (e.g., superoxide dismutase, catalase), and preserving redox homeostasis. Importantly, insulin itself exhibits anti-inflammatory and anti-oxidative properties—it suppresses the generation of free radicals, inhibits lipid peroxidation, and thereby modulates endothelial senescence and systemic inflammation [11,24,25,26]. The composition of fatty acids significantly influences the biosynthesis of specific eicosanoids. Polyunsaturated fatty acids (PUFAs) serve as substrates for enzymatic oxidation mediated by lipoxygenases (LOXs), cyclooxygenases (COXs), and cytochrome P450 enzymes, leading to the formation of various bioactive lipid mediators. These include, among others, prostaglandins, prostacyclins (PGs), thromboxanes (TXs), leukotrienes (LTs), resolvins, maresins, hydroxyeicosatetraenoic acids (HETEs), and hydroxyoctadecadienoic acids (HODEs) [27,28,29,30]. Arachidonic acid (AA), an omega-6 PUFA, is the principal precursor for many eicosanoids such as PGE_2_, LTB_4_, and TXA_2_, which are typically associated with pro-inflammatory responses. In contrast, omega-3 PUFAs such as eicosapentaenoic acid (EPA) and docosahexaenoic acid (DHA) give rise to structurally similar but functionally distinct mediators—including PGE_3_, LTB_5_, and TXA_3_—that are generally involved in anti-inflammatory or inflammation-resolving processes. The biosynthetic pathways converting the two essential dietary fatty acids—linoleic acid (LA, omega-6) and α-linolenic acid (ALA, omega-3)—into long-chain PUFAs [31,32,33] and their downstream lipid mediators are illustrated in Figure 1. In addition to enzymatic pathways, non-enzymatic free radical-induced peroxidation of PUFAs—particularly arachidonic acid—gives rise to prostaglandin-like compounds known as isoprostanes (IsoPs) [34,35,36]. Isoprostanes are considered among the most reliable and specific biomarkers of lipid peroxidation and oxidative stress. Due to their stability and specificity, IsoPs have been widely employed in studies evaluating oxidative damage. Notably, studies investigating CR have demonstrated a significant reduction in circulating and urinary IsoP levels, indicating a decrease in oxidative stress and an improvement in redox homeostasis in response to metabolic adaptation [37,38].

Moreover, during caloric restriction, the expression of genes involved in fatty acid catabolism—particularly those regulating β-oxidation and gluconeogenesis—increases significantly in hepatic and skeletal muscle tissues [11,24,41]. Concurrently, the expression of proteins associated with hepatic lipogenesis is downregulated, reflecting a metabolic shift toward enhanced lipid utilization. CR also promotes autophagy, a cellular degradation process that facilitates the breakdown of triglycerides and lipid droplets, thereby supporting lipolysis and energy homeostasis under conditions of limited nutrient availability [42]. In addition, CR has been demonstrated to exert anti-inflammatory effects, which are mechanistically linked to the downregulation of genes encoding key pro-inflammatory mediators. Specifically, CR—even when implemented intermittently, such as in the intervals between meals—leads to a decreased expression of interleukin-6 (IL-6), tumor necrosis factor alpha (TNF-α), and chemokine (C-X-C motif) ligand 2 (CXCL2) in adipose tissue. This reduction in cytokine gene expression reflects a suppression of inflammatory signaling pathways, contributing to the overall immunomodulatory and metabolic benefits associated with CR. Among the nutritional modulators of inflammation, omega-3 fatty acids are known for their strong anti-inflammatory properties, exerting inhibitory effects on IL-1β, TNF-α, and IL-6 production [43,44]. In contrast, omega-6 fatty acids are generally considered pro-inflammatory and, in the gut, have been shown to influence both microbial diversity and intestinal barrier function, thereby affecting host metabolic health. Dietary intake of these fatty acids can alter their incorporation into the membrane lipids of various tissues—including muscle, erythrocytes, and the intestinal epithelium [45,46]—ultimately reflecting the impact of nutrition on cellular lipid composition and systemic inflammation. These molecular changes are accompanied by improved insulin sensitivity, reduced hepatic steatosis, and normalization of plasma lipid parameters, including cholesterol and triglyceride levels, as demonstrated in experimental studies using C57BL/6J mice [47,48].

CR is therefore a diet used for weight reduction, but it can also be a preventive measure for healthy ageing and can influence the extension of life. Although the metabolic benefits of CR have been widely documented, its impact on lipid mediator profiles at the tissue level remains insufficiently characterized. In particular, there is a lack of detailed studies assessing how long-term CR influences the tissue concentrations of PUFAs and their downstream lipid derivatives in organs actively involved in metabolism and immunoregulation, such as the intestine and skeletal muscle. These tissues play a key role in systemic energy balance, immune signaling, and the resolution of inflammation [49,50], all of which are known to be affected by CR. Previous research has indicated that dietary interventions can alter the availability of lipid substrates and modify the pro- and anti-inflammatory balance. However, data on the tissue-specific effects of sustained caloric restriction on these lipid pathways during physiological ageing are limited and largely descriptive. There is also a need for integrative studies that do not merely catalog individual lipid species but instead examine them in the context of their biological function and metabolic relevance. Addressing this gap, the present study focuses on characterizing how long-term caloric restriction influences the levels of selected fatty acids and lipid mediators in skeletal muscle and intestinal tissues. Based on previous evidence indicating that long-term CR influences fatty acid availability and tissue-specific lipid metabolism, we hypothesized that CR induces alterations in the concentrations of PUFAs, including a reduction in essential fatty acids—LA and ALA—and their downstream metabolites, such as AA, EPA, and DHA, in both intestinal and skeletal muscle tissues. We further hypothesized that these potential changes in PUFA availability would be associated with corresponding alterations in the profiles of lipid mediators derived from these fatty acids, including resolvins, prostaglandins, and lipoxins. Given the distinct physiological and metabolic functions of the intestine and skeletal muscle, we anticipated that the observed lipidomic shifts would reflect this tissue specificity—with the intestine representing the primary interface for dietary lipid absorption and microbial interactions, and skeletal muscle exhibiting relatively minor alterations in fatty acid profiles under caloric restriction, as reported in previous studies.

## 2. Materials and Methods

### 2.1. Animals and Diets

The animal experiment was conducted at the Department of Physiology of the Pomeranian Medical University in Szczecin. Animal handling was performed in accordance with the procedures and protocols for handling approved by the Local Ethical Committee (Resolution No. 27/2012 of 17 October 2012). The number of animals used was reduced in accordance with the 3Rs principles recommended by the Ethics Committee. Experimental animals were supplied by the Center for Experimental Medicine of the Medical University in Białystok. All these data and the criteria for the inclusion and exclusion of animals in the study were specified in the application to the Local Ethical Committee in Szczecin. For the first week of the experiment, handling was conducted (every day the animals were habituated and familiarized with the presence of the experimenter). The study was performed over a duration of 8 months.

The research was conducted on 8-week-old C57BL/6 mice of both sexes. Animals were randomly assigned to the control group (C) and the experimental group (B), with 12 mice in each group (six males and six females), resulting in a total of 24 animals. There were no significant differences in body weight between the groups at baseline. Throughout the 8-month experimental period, mice were housed individually (one mouse per cage) in a controlled environment maintained at 21 °C, with a 12:12 h light/dark cycle and adequate ventilation. Wood-shaving bedding was used, and relative humidity was maintained at 40–60%, in accordance with the EU Directive 2010/63/EU on the protection of animals used for scientific purposes. The control groups (C1—males and C2—females) received a standard feed pellet for a period of eight months—Labofeed (Poland), in the amount consistent with the recommended daily dose (7 g per mouse). The daily food intake among the study groups (B1—males and B2—females) was reduced by 30% (calorie restriction diet—Every Other Day feeding (EOD)) [51], which is used worldwide in many research centers [21,52,53]. All animals had free access to water (ad libitum). Body weight was measured once a week, beginning with day 0.

### 2.2. Tissue Preparation

At the end of the experimental period, animals were anesthetized by intravenous administration of thiopental at a dose of 90 mg/kg body weight [54]. Following the confirmation of deep anesthesia, euthanasia was performed by exsanguination. Tissue samples were immediately collected and stored at −80 °C for further analysis.

Skeletal muscle tissue from the thigh (hindlimb) region and samples of the large intestine (colon) were collected for subsequent analyses. Each tissue was homogenized in liquid nitrogen prior to analysis. Briefly, tissues were immersed in liquid nitrogen and subsequently subjected to mechanical impact (manual hammering), ensuring complete disruption while maintaining low temperature to prevent lipid degradation.

### 2.3. The Analysis of Fatty Acids Derivatives

#### 2.3.1. Reagents

Standards of fatty acid derivatives were used for the analyses: resolvin E1, resolvin D1, LXA4 5S, 6R, LXA4 5S, 6R, 15R, merazine 1, LTB4, PGE2, 10S17R-DiHDHA, 12S-HETE, 13S-HODE, 15S-HETE, 16RS-HETE, 17RS-HDHA, 18RS-HEPE, 5-HETE, 5oxo-ETE, 9S-HODE, lipoxin A4 (Sigma-Aldrich, Merck KGaA, Darmstadt, Germany), and PGB2, (Cayman, cat no. 11210). The corresponding reagents were methanol (VWR, Gdańsk, Poland), acetic acid (Sigma-Aldrich, Merck KGaA, Darmstadt, Germany), acetonitrile (VWR, Gdańsk, Poland), buffered sodium chloride solution (PBS, Sigma-Aldrich, Merck KGaA, Darmstadt, Germany), ethyl acetate (Honeywell, Boston, MA, USA), and hydrochloric acid (VWR, Gdańsk, Poland).

#### 2.3.2. The Isolation of Fatty Acid Derivatives

Based on our predefined hypotheses, the primary outcome of this study was the quantification of PUFA-derived lipid mediators in the intestinal and skeletal muscle tissues of mice subjected to long-term caloric restriction. Analysis of fatty acid derivatives was performed from muscle and intestinal tissues collected from the animals. These tissues were first homogenized, and then 150 μL of PBS was added. 50 μL of PGB2 was used as an internal standard. After adding 1 mL of 99.9% acetonitrile to the samples, the samples were incubated for 10 min at minus 20 °C and centrifuged at 1617 RCF, for 20 min at 4 °C. 4.5 mL of 1 mM hydrochloric acid and 50 μL of 1 M hydrochloric acid were also added to the samples. Isolation was then carried out using Bond-Elut C18 columns (Agilent Technologies, Lexington, MA, USA), which were first activated by adding 2.5 mL of 100% acetonitrile and 2.5 mL of 20% acetonitrile in 1 mM hydrochloric acid. After the sample supernatants were applied to the columns, 3 mL of a 20% acetonitrile solution in 1 mM hydrochloric acid was passed through the columns. Finally, they were eluted from the columns with 1.5 mL of a methanol/ethyl acetate mixture (1:1). They were subsequently dried under vacuum, and 100 µL of 0.01% acetic acid in methanol was added to the resulting precipitate. The prepared samples were subjected to chromatographic analysis.

#### 2.3.3. Liquid Chromatography Analysis

Agilent Technologies 1260 HPLC System (Agilent Technologies, Santa Clara, CA, USA), liquid chromatography was used for the analysis of fatty acid derivatives. The flow rate through the Poroshell 120 SBC 18 column used for separation (3 mm × 100 mm × 2.7 μm) was set at 1 mL/min. Two solutions were used for the analysis: solution A (99.9% methanol/0.1% acetic acid) and solution B (50% methanol/50% H_2_O/0.1% acetic acid). Initially, the separation took place under the conditions of solution A 90% (0 min), then the gradient changed to 15% solution A (12.5 min), and, finally, the 100% concentration of solution B was maintained for 20 min.

The diode array detector (DAD) monitored chromatographic peaks at wavelengths of 235 nm, 280 nm, and 210 nm. Fatty acids were identified by comparing their retention times with those of commercially available standards. The relative concentrations of individual fatty acids were calculated with reference to the internal standard—PGB_2_. Calibration curves were constructed using commercially available standards over concentration ranges relevant to the expected tissue levels. The applied method for the analysis of fatty acid derivatives followed previously published protocols [55,56].

### 2.4. The Analysis of Fatty Acids

#### 2.4.1. Reagents

Standards of fatty acid were used for the analyses: decanoic acid (C10:0), undecanoic acid (C11:0), lauric acid (C12:0), myristic acid (C14:0), myristoleic acid (C14:1), pentadecylic acid (C15:0), palmitic acid (C16:0), palmitoleic acid (C16:1), margaric acid (C17:0), heptadecenoic acid (C17:1), stearic acid (C18:0), vaccenic acid (C18:1*n*-7), oleic acid (C18:1*n*-9), linoleic acid (C18:2*n*-6), γ-linolenic acid (C18:3*n*-6, GLA), alpha-linolenic acid (C18:3*n*-3), arachidic acid (C20:0), eicosenoic acid (C20:1), eicosatrienoic acid (C20:3*n*-6), arachidonic acid (C20:4*n*-6, AA), eicosapentaenoic acid (C20:5*n*-3), behenic acid (C22:0), erucic acid (C22:1*n*-9), docosatetraenoic acid (C22:4*n*-6), docosapentaenoic acid (C22:5*n*-3), docosahexaenoic acid (C22:6*n*-3), tricosanoic acid (C23:0), lignoceric acid (C24:0), nervonic acid (C24:1) (Sigma-Aldrich, Merck KGaA, Darmstadt, Germany), and internal standard—heneicosanoic acid (C21:0) (Merck, Darmstadt, Germany). The corresponding reagents were chloroform (VWR, Gdańsk Poland), methanol (VWR, Gdańsk Poland), butylated hydroxytoluene (BHT, Sigma-Aldrich, Merck KGaA, Darmstadt, Germany), 14% boron trifluoride in methanol (VWR, Gdańsk Poland), potassium hydroxide (Sigma-Aldrich, Merck KGaA, St. Louis, MI, USA, and Burlington, MA, USA), sodium chloride (Thermo Fisher Scientific, Waltham, MA, USA), and hexane (Sigma-Aldrich, Merck KGaA, St. Louis, MI, USA, and Burlington, MA, USA). The separation was performed using a capillary column with dimensions of 15 m × 0.10 mm and a film thickness of 0.10 μm (Supelcowax™ 10 Capillary GC Column, Supelco, Bellefonte, PA, USA).

#### 2.4.2. The Isolation of Fatty Acid

The tissue-specific profiles of individual fatty acids were assessed as secondary outcomes, to explore potential changes in PUFA availability that could underlie shifts in lipid mediator biosynthesis. Fatty acid methyl esters were isolated from homogenized skeletal muscle and intestinal tissue. To obtain fatty acid esters, muscle and intestinal homogenates were isolated using a modified Folch method [57]. First, chloroform and methanol (2:1 ratio) were added to the samples, followed by addition of 100 µL internal standard, which was C21 (concentration of 2 mg/mL), and 50 µL of the BHT solution (0.5% 2,6-di-t-butyl-cresol). Then, the samples were mixed well using a horizontal shaker for 20 min, and, after this time, they were centrifuged in a centrifuge at 10 min at 1008 RCF. To the obtained supernatant, 1 mL of 2 M of a solution of potassium hydroxide in methanol was added, and the sample was heated for 20 min at ~70 °C. After incubation, 1 mL of a 14% solution of boron trifluoride in methanol was added to the samples, and incubation was repeated. The resulting fatty acid esters were dissolved in 2 mL of hexane, after adding 10 mL of saturated NaCl solution to the samples. Hexane was transferred to chromatographic vials and analyzed for fatty acid profile.

#### 2.4.3. Gas Chromatography Analysis

The fatty acid ester profile was determined by gas chromatography. Gas chromatography was performed using the Agilent Technologies 7890A GC System, Santa Clara, CA, USA. Chromatographic conditions were as follows: the initial temperature was 60 °C for 0 min, increased at a rate of 40 °C/min to 160 °C (0 min), increased at a rate of 30 °C/min to 190 °C (0.5 min), and then increased at a rate of 30 °C/min to 230 °C for 2.6 min, where it was maintained for 4.9 min. The total analysis took about 8 min, and the gas flow rate was 0.8 mL/min with hydrogen as the carrier gas.

The analytical procedure was based on methods widely accepted in lipidomic studies [30,55]. Fatty acids were qualitatively identified by comparing their retention times with those of commercially available standards. Quantitative analysis was performed using ChemStation Software B.04.03 (Agilent Technologies, Cheshire, UK; Santa Clara, CA, USA). The relative concentrations of individual fatty acids were calculated with reference to the internal standard (C21:0), ensuring reliable relative quantification.

### 2.5. Statistical Analysis

Statistical analysis was carried out using Statistica versions 13.3 (TIBCO Software Inc., Palo Alto, CA, USA) or Python (v. 3.10) in PyCharm Professional 2025.3.1.1 IDE (JetBrains, Prague, Czech Republic). First, the Shapiro–Wilk test was used to check the normality of the distribution, and then non-parametric tests were used for calculations. As the majority of variables deviated from normality, results are presented as median and interquartile range (IQR), and non-parametric or rank-based methods were used for inference. Comparisons between the control and caloric restriction groups were conducted using two-sided Mann–Whitney U tests. In addition, sex was treated as a biological variable by applying a two-factor factorial model with diet group (control vs. caloric restriction) and sex (male vs. female). Inference for main effects and interaction was based on ranks (rank-based ANOVA). To control the false discovery rate (FDR) arising from testing multiple lipid species, *p*-values were adjusted using the Benjamini–Hochberg procedure within each analyte class and tissue. Both raw and FDR-adjusted *p*-values (q-values) are reported. Statistical significance was defined as q < 0.05; unadjusted *p*-values are provided for transparency. For exploratory outcomes, effect estimates and adjusted *p*-values are reported descriptively.

## 3. Results

At baseline, the body weight of animals did not differ significantly between the control and experimental groups within each sex (Table 1). After the experimental period, both male and female mice in the control group showed a greater increase in body weight compared to their counterparts in the experimental group. In males, body weight increased by 45.81% in the control group and by 21.20% in the experimental group (*p* < 0.05). In females, the control group gained 41.85%, while the experimental group gained 31.57%; however, this difference was not statistically significant.

In muscle tissue, several statistically significant changes were observed in lipid mediators following long-term CR, as compared to the control group. After correction for multiple testing (FDR-adjusted q-values) and accounting for sex as a biological variable, five lipid mediators remained significantly lower in the CR group: resolvin E1 (0.006 ng/mL vs. 0.011 ng/mL; *p*-adj < 0.05, q-adj < 0.05), resolvin D1 (0.004 vs. 0.008 ng/mL; *p*-adj < 0.05, q-adj < 0.05), LXA4 (5S,6R) (0.028 vs. 0.042 ng/mL; *p*-adj < 0.05, q-adj < 0.05), 13S-HODE (0.057 vs. 0.216 ng/mL; *p*-adj < 0.05, q-adj < 0.05), and 9S-HODE (0.057 vs. 0.168 ng/mL; *p*-adj < 0.05, q-adj < 0.05). Other mediators, such as PGE2, LXA4 (5S,6R,15R), LTB4, maresin 1, 10S17R-DiHDHA, 18RS-HEPE, 16RS HETE, 15S HETE, 17RS HDHA, 12S HETE, 5 HETE, and 5oxo ETE did not differ significantly between groups. The results of the fatty acid derivative analysis are shown in Table 2 for muscle tissue.

In intestinal tissue, only one lipid mediator, 17RS-HDHA, remained significantly altered after FDR correction and accounting for sex as a biological variable. Specifically, its concentration was significantly lower in the CR group compared to controls (0.204 vs. 0.560 ng/mL; *p*-adj < 0.05, q-adj < 0.05). While other lipid mediators such as resolvin E1, LXA4 (5S,6R), maresin 1, 13S-HODE, and 9S-HODE showed reductions in the CR group at the unadjusted level, these differences did not retain statistical significance after adjustment for multiple comparisons and sex effects. No significant results were detected in mediators such as PGE2, resolvin D1, LXA4 (5S,6R,15R), 10S17R DiHDHA, LTB4 18RS HEPE, 16RS HETE, 15S HETE, 12S HETE, 5 HETE, and 5oxo ETE. Full results for intestinal lipid mediators are presented in Table 3.

In skeletal muscle tissue, no statistically significant changes in fatty acid composition were observed after applying FDR correction and including sex as a biological variable, with the exception of a modest reduction in docosahexaenoic acid (C22:6*n*-3, DHA) levels in the CR group compared to controls (0.744 ng/mL vs. 1.040 ng/mL; *p*-adj < 0.05, q-adj > 0.05). However, this difference did not meet the predefined significance threshold of q < 0.05 after adjustment for multiple comparisons. Similarly, although pentadecanoic acid (C15:0) levels appeared lower in the CR group (0.038 ng/mL vs. 0.058 ng/mL; unadjusted *p* < 0.05), the effect was not significant after FDR correction (q-adj > 0.05). Thus, no fatty acids remained significantly altered under caloric restriction after rigorous statistical control for multiple testing. The detailed results are presented in Table 4.

In intestinal tissue, the comparison between the control and CR groups revealed minimal statistically significant differences in fatty acid composition after correction for multiple testing (FDR-adjusted q-values) and consideration of sex as a biological variable. Only two fatty acids—linoleic acid (C18:2*n*-6) and eicosenoic acid (C20:1)—demonstrated unadjusted *p*-values below 0.05, with decreased concentrations observed in the CR group. However, these differences did not remain statistically significant following FDR correction (q > 0.5), nor did they retain significance after adjusting for sex (q-adj > 0.5). No fatty acids exhibited statistically significant differences after adjustment. The results suggest that, despite minor trends toward reduction in selected fatty acids, the changes did not reach statistical significance under rigorous multiple testing control. These findings are summarized in Table 5.

## 4. Discussion

This study aimed to investigate the effects of long-term CR on fatty acid composition and lipid mediator profiles in skeletal muscle and intestinal tissue, with a particular emphasis on PUFAs and their downstream bioactive derivatives. While numerous studies have described CR-induced alterations in membrane lipid composition—particularly within phospholipid fractions such as phosphatidylcholine, sphingomyelin, and lysophosphatidylcholine [58,59]—less is known about how prolonged energy restriction impacts the profiles of lipid mediators derived from these fatty acids. To address this knowledge gap, we characterized tissue-specific levels of selected bioactive lipid mediators in animals subjected to long-term CR. Our primary hypothesis proposes that CR would reduce tissue concentrations of fatty acids, mainly essential fatty acids—LA and ALA—as well as their long-chain derivatives, including AA, EPA, and DHA, ultimately leading to decreased biosynthesis of PUFA-derived lipid mediators. Moreover, given the distinct physiological and metabolic functions of the intestine and skeletal muscle, we further hypothesized that these changes would display tissue specificity, with more pronounced alterations in intestinal tissue due to its central role in dietary lipid absorption and interaction with the gut microbiota.

In our study, despite CR-induced reductions in certain precursor PUFAs, the concentrations of most lipid mediators—particularly in intestinal tissue—did not remain statistically significant after correction for multiple comparisons, except for 17RS-HDHA. Therefore, the initial hypothesis—that long-term caloric restriction would induce measurable shifts in lipid profiles, particularly in intestinal tissue—was only partially supported by the data. This suggests that the study, as currently designed, lacked sufficient statistical power to confirm these trends. Increasing the number of animals per group in future experiments would improve the statistical power needed to confirm these effects. Thus, the current findings should be interpreted as hypothesis-generating and call for validation in larger, adequately powered studies. Moreover, the observed changes in PUFA-derived lipid mediators under CR conditions should be interpreted in the context of well-characterized enzymatic competition between omega-3 and omega-6 PUFAs. Both LA- and ALA-derived PUFAs require the same enzymes—namely elongases and desaturases—for conversion into their respective long-chain derivatives, which ultimately serve as substrates for bioactive lipid mediator synthesis [60,61,62,63]. It is also important to note that in rodents, the metabolic conversion efficiency of essential fatty acids into long-chain PUFAs is generally higher than in humans, due to species-specific differences in enzymatic activity [46]. This may result in more dynamic shifts in tissue lipid profiles in response to dietary interventions and could partially explain the observed variability.

Also, numerous rodent studies have demonstrated that CR delays the onset of age-related alterations in skeletal muscle structure and function [9,16,17,64,65]. Previous investigations, including our own [66], have shown that long-term CR is associated with a reduction in muscle mass; however, this decrease does not necessarily lead to a decline in muscle strength, suggesting that functional capacity may be preserved despite structural remodeling [67]. Skeletal muscle is a metabolically active tissue with a key role in fatty acid oxidation and systemic energy homeostasis. Under physiological conditions, de novo lipogenesis in skeletal muscle is minimal, and the muscle lipid profile is largely shaped by dietary intake [45,46]. In our study, long-term CR resulted in only one statistically significant change in skeletal muscle fatty acid composition prior to FDR correction—a reduction in pentadecanoic acid (C15:0) levels. Although not extensively synthesized endogenously, C15:0 is an odd-chain saturated fatty acid with reported anti-inflammatory and mitochondrial-protective properties [9,68,69,70]. However, the lack of FDR-corrected significance suggests that CR had a modest overall impact on muscle lipid composition. This observation is consistent with previous reports indicating that skeletal muscle lipid profiles exhibit limited responsiveness to dietary energy restriction [45]. Given the high mitochondrial density of skeletal muscle, changes in fatty acid composition may also reflect oxidative remodeling processes triggered by prolonged caloric deficit. Under such conditions, increased production of reactive oxygen species (ROS) can promote lipid peroxidation through non-enzymatic pathways such as the isoprostane pathway, resulting in stable peroxidation products and potentially altering the pool of available fatty acid substrates [68,69]. However, numerous studies have shown that long-term CR mitigates oxidative stress by promoting mitochondrial biogenesis, improving mitochondrial efficiency, and reducing ROS generation [70,71,72,73]. The absence of significant changes in PUFA levels in our study may therefore reflect a protective metabolic adaptation that preserves membrane lipid integrity despite energy restriction. These mitochondrial adaptations are accompanied by a downregulation of pro-oxidative and pro-inflammatory gene expression, as previously demonstrated in our own studies [74]. Furthermore, CR may help preserve the regenerative capacity of skeletal muscle by maintaining the pool of satellite cells—myogenic progenitor cells essential for growth and repair—which decline with age [75,76]. Notably, previous studies have demonstrated that certain unsaturated fatty acids can modulate satellite cell proliferation and differentiation, influencing muscle regenerative potential. These findings raise the possibility that CR-induced changes in fatty acid availability may also indirectly affect muscle repair processes via satellite cell regulation [77]. Additionally, alterations in gut microbiota composition—known to occur under caloric restriction—can influence the endogenous production and intestinal absorption of odd-chain fatty acids such as C15:0 [78]. This highlights the complexity of interpreting tissue-specific lipidomic changes, which may result from a combination of systemic metabolic adaptations and microbiota-mediated mechanisms, rather than direct dietary effects alone.

It should also be emphasized that the intestinal mucosa represents a highly metabolically active and immunologically dynamic environment, playing a central role in nutrient absorption, lipid metabolism, immune surveillance, and systemic inflammatory regulation [60,79,80,81,82]. Due to its high cellular turnover and constant exposure to both dietary and microbial stimuli, the intestinal epithelium is particularly susceptible to changes in lipid availability and composition. Indeed, the intestine is widely recognized as one of the most sensitive sites for detecting lipidomic alterations in response to dietary interventions. The absorption of long-chain fatty acids (LCFAs) is closely linked to the structural and functional integrity of the intestinal barrier, which separates luminal contents from systemic circulation and contributes to immune homeostasis [44]. Additionally, medium-chain fatty acids (MCFAs) are efficiently taken up by enterocytes and rapidly utilized for energy production, thus supporting mucosal integrity and epithelial function [44]. The composition of dietary fatty acids has also been shown to profoundly influence intestinal immune responses and modulate the development and progression of various gastrointestinal diseases, including inflammatory bowel disease, enteric infections, and colorectal cancer [43,83]. Although we anticipated robust alterations in the intestinal fatty acid profile under caloric restriction—particularly due to the gut’s metabolic plasticity—these expectations were not fully met. As previously noted, the statistical significance of the observed reductions in linoleic acid (LA) and eicosenoic acid (C20:1) did not persist after multiple testing correction. LA is a key dietary ω-6 PUFA and serves not only as a fundamental component of membrane phospholipids but also as a precursor for lipid mediators involved in epithelial homeostasis, including regulators of barrier function, immune responses, and cell proliferation—partly through PPARγ activation and NF-κB inhibition [26,79]. Thus, reduced LA availability may theoretically impair the intestinal epithelium’s ability to mount adequate responses to inflammatory stimuli. Our previous research has shown that similar reductions in LA and other PUFAs under CR conditions can occur in hepatic tissue [84], suggesting a systemic trend in fatty acid modulation. It should also be acknowledged that, while storage of samples at −80 °C is a widely accepted practice in lipidomics, it may influence the stability of certain highly unsaturated fatty acids. Nevertheless, we did not observe the expected changes, which may reflect a combination of dietary restriction, altered microbial metabolism, and tissue-specific lipid turnover, rather than sample degradation alone.

A growing body of evidence links dietary restriction to remodeling of the gut microbiota, which in turn modulates host lipid metabolism. Fiber-fermenting bacteria and bile acid-modifying microorganisms have been shown to influence intestinal PUFA availability and determine their metabolic fate. Microbiota-derived metabolites can regulate the expression of intestinal elongases and desaturases, as well as the activity of LOX and COX enzymes involved in the biosynthesis of lipid mediators. Although our study did not directly assess gut microbiota composition, gene expression, or enzymatic activity of LOX or COX isoforms, such mechanisms remain plausible contributors to the observed lipidomic alterations. In intestinal tissue, most changes in eicosanoid concentrations did not remain statistically significant following multiple testing correction, with the exception of 17RS-HDHA, which retained significance after accounting for sex as a biological variable. In turn, although, in skeletal muscle, no substantial changes were observed in total fatty acid composition, a significant reduction was noted in several PUFA-derived lipid mediators after FDR adjustment and inclusion of sex. These included resolvin E1, resolvin D1, LXA_4_ (5S,6R), 13S-HODE, and 9S-HODE. These mediators are biosynthesized through enzymatic oxidation pathways—predominantly 5-LOX and 15-LOX—and are centrally involved in the resolution of inflammation, tissue repair, and homeostatic regulation [85,86,87]. Although our study did not include direct assessment of LOX expression or activity, the observed decline in these anti-inflammatory mediators under long-term CR may reflect several non-mutually exclusive mechanisms, including decreased precursor availability (e.g., reduced tissue levels of LA or DHA, which showed non-significant downward trends), altered degradation or turnover kinetics of lipid mediators, and a metabolic reprogramming under CR that suppresses energetically costly biosynthetic processes, such as lipid mediator production, in favor of energy conservation. The observed decrease in 17RS-HDHA, a DHA-derived lipid mediator, further suggests that both ω-6 and ω-3 PUFA pathways may be affected by prolonged caloric restriction. Notably, no compensatory increases were observed in pro-inflammatory mediators such as PGE or LTB4, supporting the hypothesis that long-term CR does not simply suppress resolution signaling but may broadly downregulate eicosanoid synthesis. Such metabolic adaptations are consistent with the known effects of CR, which include enhanced mitochondrial function, increased β-oxidation, and reduced ROS production—all of which may contribute to redox homeostasis but potentially at the expense of lipid signaling capacity. These results align with our previous findings in hepatic tissue, where long-term CR was associated with reduced oxidative stress and modulation of redox-sensitive gene expression [88]. Mechanistically, lipid mediator biosynthesis follows a biphasic model: during acute inflammation, AA is mobilized from membrane phospholipids and converted into pro-inflammatory eicosanoids such as PGE2 and LTB4. In the resolution phase, distinct enzymatic cascades generate pro-resolving mediators—including lipoxins, resolvins, protectins, and maresins—that terminate inflammation and restore tissue integrity [39,89]. Impairment of this transition, whether due to reduced precursor availability, altered expression or activity of phospholipases (e.g., cPLA_2_, iPLA_2_, sPLA_2_), or LOX isoform suppression, may compromise resolution capacity during prolonged energy restriction [39,40,79].

Notably, ageing itself promotes increased oxidative stress and alters PUFA composition, often resulting in a shift towards more peroxidation-prone lipid species due to reduced anti-oxidant capacity [90,91]. Given the 8-month duration of the intervention in our study—equivalent to a significant portion of the murine lifespan—our results likely reflect not only the direct effects of long-term CR but also age-associated metabolic remodeling. Prior studies have demonstrated that even moderate CR can induce transcriptional and epigenetic reprogramming affecting key metabolic pathways, including gluconeogenesis, fatty acid oxidation, and lipid remodeling [11,24,41,59]. These systemic adaptations could influence the availability of fatty acid precursors and the enzymatic efficiency of lipid mediator production.

However, we would like to emphasize once again that, while our findings are consistent with this model, the lack of direct molecular data on enzyme expression or signaling activity limits mechanistic interpretation. Our study did not include transcriptomic or proteomic assessments, nor did it assess enzyme activity or histopathology, limiting our ability to draw mechanistic conclusions. Therefore, future studies incorporating transcriptomic, enzymatic, histological, and microbiome profiling are warranted to clarify whether the observed suppression of lipid mediator biosynthesis under CR reflects reduced substrate availability, altered microbial metabolism, or host enzymatic downregulation. An adequate sample size will also be required to reliably estimate these mechanisms and distinguish true biological variation from statistical noise.

## 5. Conclusions and Limitations

In this exploratory study, we investigated the impact of long-term caloric restriction on tissue-specific lipidomic profiles, with a particular focus on polyunsaturated fatty acids (PUFAs) and their downstream bioactive lipid mediators in skeletal muscle and intestinal tissue. Although several reductions in essential fatty acids and PUFA-derived mediators were observed, only a limited number of these changes remained statistically significant after correction for multiple comparisons. Notably, 17RS-HDHA in the intestine and lipid mediators in skeletal muscle—including resolvin E1, resolvin D1, 5S,6R-LXA_4_, 13S-HODE, and 9S-HODE—retained significance when sex was accounted for as a biological covariate. These findings suggest that prolonged caloric restriction may alter both ω-3 and ω-6 PUFA-derived resolution pathways. However, no compensatory increases in pro-inflammatory eicosanoids were detected, supporting the hypothesis that long-term caloric restriction may suppress lipid mediator biosynthesis globally rather than shift it toward a pro-inflammatory profile. These observations align with established models of caloric restriction–induced metabolic reprogramming, which prioritize oxidative metabolism and redox homeostasis over biosynthetic and inflammatory processes. The intestine and skeletal muscle appear to respond differently to prolonged energy restriction, which may reflect their distinct metabolic roles, exposure to the microbiota, and baseline lipid turnover rates.

Despite these observations, the conclusions drawn from this study are subject to several important limitations. First, the statistical power was limited due to the relatively small sample size, which may have contributed to the loss of significance for several biologically relevant findings after FDR correction. Future studies with larger cohorts will be necessary to robustly detect and validate subtle but physiologically meaningful changes in lipid mediator levels. Second, while the data support alterations in lipid mediator profiles, the absence of complementary transcriptomic, proteomic, enzymatic, and histopathological analyses restricts the ability to determine underlying mechanisms. Specifically, we did not assess the expression or activity of key enzymes involved in lipid metabolism (e.g., LOX, COX, PLA_2_ isoforms) or evaluate gut microbiota composition, which are likely to influence tissue lipid homeostasis under caloric restriction. Third, the study was performed in a murine model, which—although valuable for mechanistic insights—may not fully replicate human lipid metabolism. Rodents exhibit a higher enzymatic capacity for long-chain PUFA synthesis compared to humans, which may exaggerate certain effects of dietary interventions.

In conclusion, our findings provide initial evidence that long-term caloric restriction modulates lipid mediator profiles in a tissue-specific manner. These results should be interpreted as hypothesis-generating and call for further studies incorporating comprehensive multiomic approaches, histopathological validation, and adequate statistical power to elucidate the mechanisms by which caloric restriction shapes tissue lipid signaling networks.

## Figures and Tables

**Table 1:** Changes in body weight before and after the experimental period in male and female mice (* *p* < 0.05).

Sex	Group	Body Weight Before (g)	Body Weight After (g)	Body Weight Gain (%)
Males	Control group	28.66 ± 0.75	41.79 ± 4.96	45.81% *
	Study group	24.48 ± 1.47	29.67 ± 2.54	21.20% *
Females	Control group	21.24 ± 1.15	30.13 ± 3.35	41.85%
	Study group	22.14 ± 1.97	29.13 ± 2.46	31.57%

**Table 2:** Content of fatty acid derivatives (ng/mL) in muscle tissue between control and study group.

Variable	Group Control		Study Groups		*p*	q	*p*-adj	q-adj
	Median	IQR	Median	IQR				
resolvin E1	0.011	0.006	0.006	0.006	0.014	0.065	0.004	0.029
PGE2	0.042	0.053	0.020	0.024	0.159	0.266	0.135	0.229
resolvin D1	0.008	0.006	0.004	0.003	0.006	0.065	0.002	0.029
LXA4 (5S,6R)	0.042	0.051	0.028	0.027	0.017	0.065	0.015	0.051
LXA4 (5S,6R,15R)	0.023	0.021	0.012	0.005	0.069	0.198	0.050	0.141
10S17R DiHDHA	0.014	0.006	0.025	0.039	0.159	0.266	0.171	0.243
maresin 1	0.007	0.007	0.004	0.004	0.539	0.602	0.567	0.643
LTB4	0.007	0.005	0.004	0.003	0.346	0.443	0.361	0.471
18RS HEPE	0.013	0.069	0.009	0.020	0.254	0.352	0.102	0.195
16RS HETE	0.286	0.326	0.066	0.129	0.107	0.258	0.065	0.157
13S HODE	0.216	0.323	0.057	0.082	0.007	0.065	0.005	0.029
9S HODE	0.168	0.317	0.057	0.072	0.011	0.065	0.008	0.036
15S HETE	0.558	0.817	0.367	0.272	0.123	0.258	0.103	0.195
17RS HDHA	0.060	0.118	0.061	0.039	0.539	0.602	0.551	0.643
12S HETE	0.265	0.465	0.229	0.210	0.228	0.344	0.148	0.229
5 HETE	0.090	0.065	0.077	0.059	0.628	0.660	0.681	0.721
5oxo ETE	0.210	0.538	0.231	0.157	0.674	0.668	0.721	0.721

**Table 3:** Content of fatty acid derivatives (ng/mL) in intestinal tissue between control and study group.

Variable	Control Group		Study Group		*p*	q	*p*-adj	q-adj
	Median	IQR	Median	IQR				
resolvin E1	0.037	0.090	0.012	0.027	0.053	0.156	0.017	0.062
PGE2	0.210	0.203	0.104	0.074	0.211	0.318	0.200	0.309
resolvin D1	0.020	0.048	0.012	0.014	0.356	0.455	0.362	0.456
LXA4 (5S,6R)	0.219	0.182	0.076	0.143	0.035	0.156	0.016	0.062
LXA4 (5S,6R,15R)	0.032	0.046	0.013	0.039	0.113	0.189	0.073	0.138
10S17R DiHDHA	0.063	0.074	0.033	0.026	0.356	0.455	0.366	0.456
maresin 1	0.040	0.031	0.014	0.022	0.053	0.156	0.060	0.138
LTB4	0.016	0.030	0.013	0.013	0.720	0.758	0.740	0.786
18RS HEPE	0.085	0.247	0.048	0.151	0.113	0.189	0.064	0.138
16RS HETE	0.224	0.150	0.211	0.219	0.968	0.967	0.931	0.931
13S HODE	1.761	1.880	0.289	0.591	0.028	0.156	0.018	0.062
9S HODE	1.328	1.522	0.225	0.386	0.017	0.156	0.009	0.062
15S HETE	4.526	3.419	1.802	1.428	0.095	0.189	0.068	0.138
17RS HDHA	0.560	0.450	0.204	0.299	0.006	0.135	0.001	0.017
12S HETE	1.372	1.592	1.729	1.704	0.400	0.475	0.375	0.456
5 HETE	0.402	0.310	0.213	0.294	0.113	0.189	0.091	0.154
5oxo ETE	0.790	0.882	0.758	0.972	0.661	0.740	0.669	0.758

**Table 4:** Content of fatty acid (ng/mL) in muscle tissue between control and study group.

Variable	Group Control		Study Groups		*p*	q	*p*-adj	q-adj
	Median	IQR	Median	IQR				
C10:0	0.318	0.209 to 0.410	0.354	0.298 to 0.410	0.644	0.838	0.404	0.692
C11:0	0.007	0.00504 to 0.00781	0.006	0.00491 to 0.00790	0.598	0.956	0.653	0.921
C12:0	0.018	0.0123 to 0.0378	0.021	0.0127 to 0.0317	0.947	0.931	0.591	0.886
C14:0	0.351	0.172 to 0.585	0.347	0.167 to 0.646	0.843	0.974	0.950	0.951
C14:1	0.056	0.0153 to 0.0684	0.027	0.0143 to 0.0623	0.356	0.931	0.570	0.886
C15:0	0.058	0.0382 to 0.130	0.038	0.0262 to 0.0567	0.041	0.974	0.854	0.951
C16:0	6.178	4.915 to 10.416	5.819	3.888 to 10.756	0.947	0.747	0.369	0.692
C16:1	2.664	1.347 to 4.119	1.359	0.786 to 2.848	0.291	0.667	0.033	0.601
C17:0	0.073	0.0452 to 0.105	0.063	0.0433 to 0.0883	0.323	0.667	0.095	0.657
C17:1	0.072	0.0448 to 0.184	0.049	0.0195 to 0.0806	0.147	0.974	0.951	0.951
C18:0	1.885	1.726 to 2.722	2.687	1.898 to 3.728	0.129	0.718	0.307	0.692
C18:1 n7	0.021	0.0130 to 0.0302	0.014	0.00857 to 0.0174	0.065	0.718	0.325	0.692
C18:1 n9	7.432	4.273 to 13.651	6.516	2.985 to 12.784	0.792	0.703	0.159	0.692
C18:2 n6	2.719	1.910 to 9.453	2.553	1.338 to 3.736	0.323	0.703	0.128	0.657
C18:3 n3	0.314	0.204 to 1.084	0.358	0.182 to 0.948	0.692	0.974	0.808	0.951
C18:3 n6	0.017	0.00667 to 0.0373	0.012	0.00981 to 0.0157	0.692	0.667	0.059	0.657
C18:4	0.026	0.0181 to 0.0535	0.020	0.0111 to 0.0317	0.262	0.718	0.346	0.692
C20:0	0.041	0.0286 to 0.0776	0.063	0.0334 to 0.0984	0.210	0.807	0.463	0.758
C20:1	0.044	0.0221 to 0.0941	0.032	0.0203 to 0.0397	0.291	0.956	0.715	0.921
C20:3 n6	0.084	0.0542 to 0.163	0.066	0.0496 to 0.101	0.391	0.956	0.712	0.921
C20:4 n6	0.478	0.387 to 0.760	0.536	0.350 to 0.726	0.895	0.718	0.283	0.692
C20:5 n3	0.027	0.0154 to 0.0940	0.026	0.0197 to 0.0417	0.947	0.718	0.216	0.692
C22:0	0.055	0.0306 to 0.0682	0.043	0.0310 to 0.0630	0.742	0.718	0.277	0.692
C22:1 n9	0.067	0.0310 to 0.0776	0.046	0.0302 to 0.0675	0.291	0.718	0.292	0.692
C22:4 n6	0.029	0.0204 to 0.0370	0.022	0.0173 to 0.0316	0.262	0.776	0.366	0.692
C22:5 n3	0.150	0.110 to 0.237	0.149	0.101 to0.251	0.895	0.974	0.899	0.951
C22:6 n3	1.040	0.570 to 1.274	0.744	0.457 to 1.056	0.114	0.667	0.015	0.544
C23:0	0.033	0.0285 to 0.0361	0.038	0.0331 to 0.0429	0.087	0.974	0.947	0.951
C24:0	0.023	0.0131 to 0.0542	0.022	0.0160 to 0.0382	0.895	0.974	0.717	0.921
C24:1	0.039	0.0217 to 0.0462	0.034	0.0216 to 0.0426	0.510	0.718	0.325	0.692

**Table 5:** Content of fatty acid (ng/mL) in intestinal tissue between control and study group.

Variable	Group Control		Study Groups		*p*	q	*p*-adj	q-adj
	Median	IQR	Median	IQR				
C10:0	0.683	0.454 to 0.929	0.835	0.621 to 1.040	0.477	0.950	0.629	0.905
C11:0	0.013	0.00955 to 0.0194	0.007	0.00574 to 0.0142	0.155	0.950	0.479	0.845
C12:0	0.043	0.0312 to 0.0592	0.049	0.0206 to 0.0727	0.929	0.731	0.182	0.654
C14:0	0.529	0.404 to 1.203	0.738	0.167 to 1.661	1.000	0.993	0.973	0.973
C14:1	0.025	0.0172 to 0.0590	0.057	0.00725 to 0.107	0.859	0.950	0.540	0.845
C15:0	0.187	0.137 to 0.304	0.116	0.0952 to 0.178	0.183	1.000	0.936	0.973
C16:0	11.255	9.332 to 20.803	12.042	2.762 to 30.245	0.859	0.993	0.887	0.973
C16:1	2.667	1.516 to 4.807	3.115	0.310 to 7.714	0.859	0.731	0.132	0.536
C17:0	0.193	0.177 to 0.289	0.137	0.0728 to 0.236	0.155	0.993	0.821	0.973
C17:1	0.144	0.0898 to 0.249	0.069	0.0267 to 0.201	0.183	0.993	0.737	0.957
C18:0	3.535	3.094 to 7.279	5.487	2.332 to 10.668	0.929	0.993	0.727	0.957
C18:1 n7	0.059	0.0232 to 0.118	0.033	0.00846 to 0.126	0.534	0.731	0.075	0.465
C18:1 n9	13.909	7.659 to 25.609	11.544	2.212 to 24.406	0.657	0.731	0.084	0.465
C18:2 n6	7.155	5.242 to 8.137	2.673	0.744 to 3.998	0.021	0.993	0.973	0.973
C18:3 n3	1.068	0.477 to 1.375	0.580	0.106 to 1.026	0.477	0.993	0.529	0.845
C18:3 n6	0.028	0.0175 to 0.0398	0.040	0.0120 to 0.0543	0.534	0.950	0.206	0.673
C18:4	0.105	0.0393 to 0.235	0.040	0.0292 to 0.0737	0.131	0.617	0.009	0.338
C20:0	0.104	0.0845 to 0.115	0.117	0.0714 to 0.273	0.594	0.731	0.069	0.465
C20:1	0.111	0.0965 to 0.153	0.063	0.0270 to 0.0891	0.033	0.950	0.466	0.845
C20:3 n6	0.245	0.136 to 0.482	0.469	0.0830 to 0.628	0.722	0.950	0.365	0.845
C20:4 n6	1.264	0.618 to 1.506	1.094	0.715 to 1.664	0.859	0.731	0.091	0.465
C20:5 n3	0.134	0.0969 to 0.174	0.144	0.105 to 0.217	0.594	0.950	0.519	0.845
C22:0	0.055	0.0306 to 0.0682	0.043	0.0310 to 0.0630	0.742	0.731	0.134	0.536
C22:1 n9	0.067	0.0310 to 0.0776	0.046	0.0302 to 0.0675	0.291	0.617	0.027	0.361
C22:4 n6	0.029	0.0204 to 0.0370	0.022	0.0173 to 0.0316	0.262	0.993	0.744	0.957
C22:5 n3	0.150	0.110 to 0.237	0.149	0.101 to0.251	0.895	0.993	0.880	0.973
C22:6 n3	1.040	0.570 to 1.274	0.744	0.457 to 1.056	0.114	0.654	0.030	0.361
C23:0	0.033	0.0285 to 0.0361	0.038	0.0331 to 0.0429	0.087	0.950	0.624	0.905
C24:0	0.023	0.0131 to 0.0542	0.022	0.0160 to 0.0382	0.895	0.950	0.436	0.845
C24:1	0.039	0.0217 to 0.0462	0.034	0.0216 to 0.0426	0.510	0.950	0.461	0.845

## Data Availability

The data are available from the corresponding author upon reasonable request.
